# Network-Dependent Modulation of COMT and DRD2 Polymorphisms in Healthy Young Adults

**DOI:** 10.1038/srep17996

**Published:** 2015-12-08

**Authors:** Fangshi Zhao, Xuejun Zhang, Wen Qin, Feng Liu, Qiuhui Wang, Qiang Xu, Junping Wang, Chunshui Yu

**Affiliations:** 1 Department of Radiology and Tianjin Key Laboratory of Functional Imaging, Tianjin Medical University General Hospital, Tianjin 300052, China; 2 School of Medical Imaging, Tianjin Medical University, Tianjin, China

## Abstract

Nonlinear modulation of the dopamine signaling on brain functions can be estimated by the interaction effects of dopamine-related genetic variations. We aimed to explore the interaction effects of COMT rs4680 and DRD2 rs1076560 on intra-network connectivity using independent component analysis. In 250 young healthy adults, we identified 11 meaningful resting-state networks (RSNs), including the salience, visual, auditory, default-mode, sensorimotor, attention and frontoparietal networks. A two-way analysis of covariance was used to investigate COMT×DRD2 interactions on intra-network connectivity in each network, controlling for age, gender and education. Significant COMT×DRD2 interaction was found in intra-network connectivity in the left medial prefrontal cortex of the anterior default-mode network, in the right dorsolateral frontal cortex of the right dorsal attention network, and in the left dorsal anterior cingulate cortex of the salience network. *Post hoc* tests revealed that these interactions were driven by the differential effects of DRD2 genotypes on intra-network connectivity in different COMT genotypic subgroups. Moreover, even in the same COMT subgroup, the modulation effects of DRD2 on intra-network connectivity were different across RSNs. These findings suggest a network-dependent modulation of the DA-related genetic variations on intra-network connectivity.

Dopamine (DA), as a critical neurotransmitter, regulates movement, cognition and reward^1^,^2^,^3^. The modulation of the DA system is realized by impacting the structure and function of the brain. The DA signaling in brain tissue is regulated by the genetic variations in the DA pathway. For example, a single-nucleotide polymorphism (SNP; Val158Met) of the catechol-O-methyltransferase (COMT) can affect the degradation of synaptic dopamine^4^,^5^ and an SNP (rs1076560, G > T) of the dopamine D2 receptor (DRD2) can affect the function of the receptor^6^. Although the DA signaling in brain tissue cannot be measured *in vivo*, it can be roughly estimated by the carrying status of the DA-related genes. For example, the DA signaling is lower in COMT Val homozygotes than in Met-carriers and lower in DRD2 GG than in TT carriers. According to status of the COMT and DRD2, we can generate subgroups with different levels of the DA signaling. By investigating the interaction effects of the two SNPs, one can *in vivo* explore how the DA system modulates brain structure and function^7^,^8^,^9^.

The human brain consists of several functional independent networks, which serves different functions. However, to date, whether and how the DA signaling modulates these functional networks remains largely unknown. Using the interaction effects of COMT and DRD2, a previous study has revealed a functional system-dependent modulation of the DA signaling on functional connectivity density (FCD) in healthy young subjects. Brain regions (temporal pole and putamen) of the “control system” and those (medial prefrontal cortex and occipital cortex) of the “processing system” have been shown to exhibit a much different modulation by the DA-related genetic variations^8^. Because the nature of the previous study is voxel-wise connectivity analysis but not a typical network analysis, the specific modulation patterns of the DA signaling on functional networks are unclear. Independent component analysis (ICA) can identify multiple resting-state networks (RSNs), which can be used to directly investigate the intra-network connectivity in each RSN^10^.

In this study, we used the ICA approach to explore the specific modulation of the DA signaling on the intra-network connectivity in the RSNs in healthy young adults by analyzing the interaction effects between the COMT rs4680 and DRD2 rs1076560.

## Materials and Methods

### Subjects

The study has been approved by the Medical Research Ethics Committee of Tianjin Medical University, and written informed consent was obtained from each subject before the study. The method was carried out in accordance with the approved guidelines. A total of 250 healthy right-handed subjects (mean age: 22.7 ± 2.4 years; 115 males) were selected from 323 Chinese Han subjects who participated in this study after giving written informed consent. Seventy-three subjects were excluded from further analysis due to a lack of genetic data (29 subjects) or excessive head movement (14 subjects) during the functional magnetic resonance imaging (fMRI) scans or missing behavioral scales (30 subjects). Careful screening was performed to ensure that all participants had no any lifetime history of psychiatric or neurological illness and MR contraindications. Memory function was evaluated with the Chinese Revised Wechsler Memory Scale^11^, and executive function was tested using the Wisconsin Card Sorting Test^12^. Individual working memory capacity was assessed using the n-back task^13^. Depression levels were examined using the Beck Depression Inventory^14^, and anxiety levels were evaluated with the Self-Rating Anxiety Scale^15^. Temperamental characteristics were assessed using the Tridimensional Personality Questionnaire^16^. These above-mentioned behavioral scales reflect structural and functional characteristics of the brain and show genotypes differences^17^,^18^,^19^,^20^,^21^.

### Genotyping

Genomic DNA was extracted from 3000 μl of whole blood using the EZgeneTM Blood gDNA Miniprep Kit (Biomiga). We determined the genotypes for COMT rs4680 and DRD2 rs1076560 of the subject using the PCR and ligation detection reaction (LDR) method^22^,^23^. The PCR primer sequences for COMT were as follows: forward: 5′ GGGCCTACTGTGGCTACTCA 3′, and reverse: 5′ CCCTTTTTCCAGGTCTGACA 3′. The PCR primer sequences for DRD2 were as follows: forward: 5′ AGCATCTCCATCTCCAGCTC 3′, and reverse: 5′ GAAAAAGGACAGGGGCAATC 3′. PCR was performed with a 20 μL reaction volume containing 1 μL genomic DNA, 0.4 μL primer mixture, 2 μL dNTPs, 0.6 μL Mg^2+^, 2 μL buffer, 4 μL Q-Solution, and 0.3 μL Taq DNA polymerase. The amplification protocol incorporates an initial denaturation and enzyme activation phase at 95°C for 15 min, followed by 35 cycles of denaturation at 94°C for 30 sec, annealing for 1 min and 30 sec at 59°C for COMT rs4680 and 56°C for DRD2 rs1076560, extension at 72°C for 1 min, and then a final extension at 72°C for 7 min. PCR products were verified in 3% agarose gels that had been stained with ethidium bromide to regulate the amount of DNA added to the LDR.

For each SNP, three probes were designed for the LDR reactions: one common probe (rs4680: P-GCCAGCGAAATCCACCATCCGCTGGTTTTTTTTTTTTTTTTTTTT-FAM; rs1076560: P-GAAAGGGAGGGGCCAGTGAGATGGGTTTTTTTTTTTTTTTTTT-FAM) and two discriminating probes for the two alleles of each SNP (rs4680_A: TTTTTTTTTTTTTTTTTTTTCAGGCATGCACACCTTGTCCTTCAT; rs4680_G: TTTTTTTTTTTTTTTTTTTTTTCAGGCATGCACACCTTGTCCTTCAC; rs1076560_T: TTTTTTTTTTTTTTTTTTGTGTTTGCAGGAGTCTTCAGAGGGA; rs1076560_G: TTTTTTTTTTTTTTTTTTTTGTGTTTGCAGGAGTCTTCAGAGGGC). These reactions were conducted in a 10 μL mixture containing 1 μL buffer, 1 μL probe mix, 0.05 μL Taq DNA ligase, 1 μL PCR product, and 6.95 μL deionized water. The reaction program consisted of an initial heating at 95°C for 2 min, followed by 35 cycles of 30 sec at 94°C and 2 min at 50°C. Reactions were stopped by chilling the tubes in an ethanol–dry ice bath and adding 0.5 mL of 0.5 mM EDTA. Aliquots of the reaction products (1 μL) were mixed with 1 μL of loading buffer (83% formamide, 8.3 mM EDTA and 0.17% blue dextran) and 1 μL ABI GS-500 Rox-Fluorescent molecular weight marker and then denatured at 95°C for 2 min.

The samples were then chilled rapidly on ice prior to being loaded on a 5 Murea-5% polyacrylamide gel and electrophoresed on an ABI 3100 DNA sequencer at 3000 V. Finally, the fluorescent ligation products were analyzed and quantified using the ABI GeneMapper software.

### Data acquisition

MRI data were acquired using a 3.0-Tesla MR system (Discovery MR750, General Electric, Milwaukee, WI, USA). Tight but comfortable foam padding was used to minimize head motion, and earplugs were used to reduce scanner noise. Resting-state fMRI data were collected using single-shot echo-planar imaging with the following imaging parameters: repetition time/echo time = 2000/30 ms; field of view = 240 mm ×240 mm; matrix = 64 × 64; flip angle = 90°, slice thickness = 4 mm; no gap; 40 interleaved transverse slices; and 180 volumes. All subjects were asked to keep their eyes closed, to think of nothing in particular and not to fall asleep, and as motionless as possible. Sagittal three-dimensional T1-weighted images were acquired by a brain volume sequence (repetition time/echo time = 8.1/3.1 ms; inversion time = 450 ms; field of view = 256 mm ×256 mm; matrix = 256 ×256; flip angle = 13°, slice thickness = 1 mm; no gap; 176 slices).

### fMRI data preprocessing

The fMRI data were preprocessed using the Statistical Parametric Mapping (SPM8, http://www.fil.ion.ucl.ac.uk/spm) and Data Processing Assistant for Resting-State fMRI (DPARSF)^24^. We discarded the first 10 volumes of each functional time series because of the signal reaching equilibrium and the participants adapting to the scanning noise. The remaining 170 volumes were corrected for the acquisition time delay between different slices. Head movement parameters were tested by estimating the translation in each direction and the angular rotation on every axis for each volume. Fourteen subjects who had a maximum displacement >2 mm or a maximum rotation >2.0° were excluded from further analysis. The movement-corrected functional volumes were spatially normalized to the Montreal Neurological Institute (MNI) space and re-sampled to 3 × 3 × 3 mm^3^ voxels using the normalization parameters estimated during the unified segmentation. Then, the resulting images were smoothed using a Gaussian kernel of 6 × 6 × 6 mm^3^ full-width at half-maximum.

### Identification of resting-state networks

We used group ICA (GICA) to decompose all the data into independent components (ICs) using the GIFT software (http://icatb.sourceforge.net/, version 1.3 h) with three steps: data reduction, ICA, and back reconstruction^25^. Data reduction was to reduce the size of the subject’s fMRI data using the principal components analysis. Two data reduction steps were adopted. After each subject’s fMRI data was reduced, the subjects were concatenated into one group and put through another data reduction step. ICA algorithm was then applied to the reduced dataset to identify ICs. The number (n = 31) of ICs was automatically estimated using the minimum description length (MDL) criterion^26^. ICASSO toolbox was used to determine the reliability of ICA algorithm. Specifically, ICA was run 100 times to obtain the final integrated output. The subject-specific time courses and spatial maps were back-reconstructed by a dual-regression method. A linear spatial regression was applied to the group-level spatial maps and individuals’ fMRI datasets to calculate matrices describing time courses for each component of each subject^25^. A linear temporal regression was then applied to these time-course matrices and individuals’ fMRI datasets to estimate subject-specific spatial maps^27^. For each subject-specific spatial component, the value of a voxel represents the relation of the time courses between this voxel and the subject-specific component. We defined this value as intra-network connectivity. To improve the normality of the data, we scaled the spatial maps to z-scores, which were used to voxel-wise compare the localized differences in the spatial component between different groups^25^,^27^. In this study, a total of 11 meaningful components were identified from the 31 ICs by visual inspection and a frequency analysis of the spectra of the estimated ICs^28^, potentially depicting functionally relevant RSNs.

### Intra-network connectivity analysis

**Figure 1:**
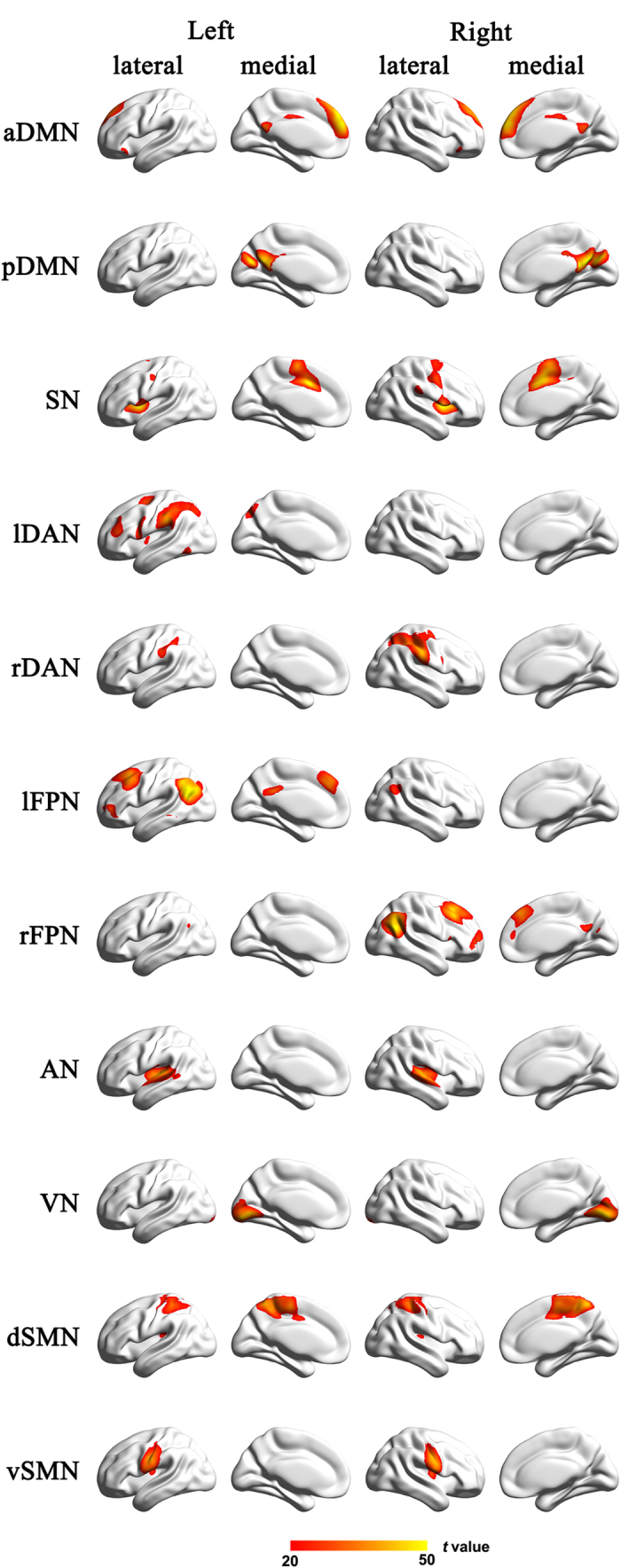
Cortical representation of the resting state networks (RSNs) identified by independent component analysis. Data are displayed on the lateral and medial surfaces of the left and right hemispheres of a brain surface map. aDMN, anterior default mode network; AN, auditory network; dSMN, dorsal sensorimotor network; lDAN, left dorsal attention network; lFPN, left frontoparietal network; pDMN, posterior default mode network; rDAN, right dorsal attention network; rFPN, right frontoparietal network; SN, salience nerwork; vSMN, ventral sensorimotor; VN, visual network. The color scale represents the *t* values in each RSN.

The eleven meaningful independent components representing RSNs were entered into a random-effect one-sample *t*-test. We used *t* > 20 and a cluster size of >100 voxels to improve the representation of each brain network. A sample-specific spatial map was generated for each component (Fig. 1). A two-way analysis of covariance (ANCOVA) was used to investigate the interaction effects between the COMT and DRD2 on intra-network connectivity in each RSN mask with gender, age and years of education as nuisance covariates. A correction for multiple comparisons was performed using a Monte Carlo simulation, resulting in a corrected threshold of *p* < 0.05 (AlphaSim program in AFNI software (http://afni.nimh.nih.gov/). Parameters: single voxel *p*= 0.05, 5000 simulations, cluster connection radius = 5 mm; with a gray matter mask and a resolution of 3 mm ×3 mm ×3 mm).

## Results

### Demographic and genetic characteristics

The demographic data are summarized in Table 1. The group of distributions in both COMT rs4680 genotypes (120 Val/Val, 106 Met/Val, and 24 Met/Met) and DRD2 rs1076560 genotypes (39 TT, 115 GT, and 96 GG) were in Hardy–Weinberg equilibrium (*p* > 0.05). Subjects with either homozygous or heterozygous for the Met-allele of COMT were merged into a group of Met-allele carriers according to previous method to address skewed genotypic distributions^19^,^29^,^30^. There was no significant interaction effect or main effect (*p* > 0.05) of COMT and DRD2 on any of the demographic, cognitive (memory and execution) and psychological (depression, anxiety, and personality) variables.

### Components of the resting-state networks

As shown in Fig. 1, eleven meaningful components were identified by visual inspection, including anterior and posterior default mode networks (aDMN and pDMN), salience network (SN), left (lDAN) and right (rDAN) dorsal attention networks, ventral (vSMN) and dorsal (dSMN) sensorimotor networks, left (lFPN) and right (rFPN) frontoparietal networks, visual network (VN), and auditory network (AN). The components and locations of these RSNs were consistent with previous studies^31^,^32^,^33^,^34^.

### Interaction effects of COMT and DRD2 on the intra-network connectivity

**Figure 2:**
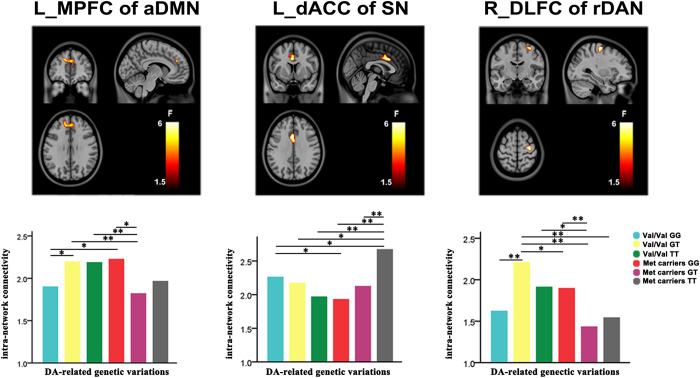
The interaction effects of COMT and DRD2 on intra-network connectivity in the brain regions (top row) of brain networks, and the intergroup differences in intra-network connectivity in each cluster of brain networks (bottom row). Color bar shows the F values. The horizontal axes of the bar plots (bottom row) represent six genotypic subgroups interacted by COMT and DRD2. The vertical axes represent the z-value of the intra-network connectivity. aDMN, anterior default mode network; dACC, dorsal anterior cingulate cortex; DLFC, dorsolateral frontal cortex; L, left; MPFC, medial prefrontal cortex; R, right; rDAN, right dorsal attention network; SN salience network. *represents p < 0.05, uncorrected for multiple comparisons; **represents p < 0.05/15 = 0.0033, Bonferroni corrected for multiple comparisons.

Although we focused on the interaction effects, we also found significant main effects of the two SNPs on intra-network connectivity (Supplementary information). The COMT × DRD2 interaction effects on intra-network connectivity was found in the left medial prefrontal cortex (MPFC) (peak MNI coordinates: *x* = −15, *y* = 45, *z* = 33) of the aDMN, in the left dorsal anterior cingulate cortex (dACC) (*x* = −3, *y* = 12, *z* = 33) of the SN, and in the right dorsolateral frontal cortex (*x* = 33, *y* = −15, *z* = 63) of the rDAN (Fig. 2 and Table 2). However, there were no significant interaction effects (*p* < 0.05, Alphasim correction) on the intra-network connectivity between these two SNPs.

*Post hoc* tests revealed that the DA-related genetic variations exhibited different nonlinear modulation patterns on intra-network connectivity in different RSNs. The intergroup differences in intra-network connectivity in the MPFC of the DMN are shown in the lower row of the left column in Fig. 2 and Table 3. In the COMT Val/Val carriers, the DRD2 GG subgroup exhibited weaker intra-network connectivity than the DRD2 GT subgroup (*p* = 0.013, uncorrected). In contrast, in the COMT Met carriers, the DRD2 GG subgroup exhibited stronger intra-network connectivity than the DRD2 GT subgroup (*p* < 0.05, Bonferroni corrected). The intergroup differences in intra-network connectivity in the DLFC of the rDAN are shown in the lower row of the middle column in Fig. 2 and Table 4. In the COMT Val/Val carriers, the DRD2 GG subgroup had weaker intra-network connectivity than the DRD2 GT subgroup (*p* < 0.05, Bonferroni corrected). In contrast, in the COMT Met carriers, the DRD2 GG subgroup showed stronger intra-network connectivity than the DRD2 GT subgroup (*p* < 0.05, Bonferroni corrected). The intergroup differences in intra-network connectivity in the dACC of the SN are shown in the lower row of the right column in Fig. 2 and Table 5. In the COMT Val/Val carriers, intra-network connectivity did not exhibit any significant differences among the DRD2 genotypic subgroups. However, in the COMT Met carriers, both the DRD2 GG and GT subgroups exhibited weaker intra-network connectivity than the DRD2 TT subgroup (*p* < 0.05, Bonferroni corrected).

## Discussion

In the present study, we investigated COMT ×DRD2 interactions on intra-network connectivity in healthy young adults. The SN, aDMN and rDAN exhibited significant COMT×DRD2 interactions that were driven by the differential effects of DRD2 genotypes on intra-network connectivity in different COMT genotypic subgroups. Moreover, even in the same COMT subgroup, the genetic modulation of the DRD2 on intra-network connectivity was different across RSNs. Because the different genotypic combinations of COMT and DRD2 may represent different presumed dopamine signaling, our findings indicate that the nonlinear modulation of the DA system on intra-network connectivity is RSN-dependent.

COMT plays a critical role in the degradation of the catecholamine neurotransmitters (noradrenaline, adrenaline and dopamine), accounting for more than 60% of the DA degradation in the prefrontal cortex due to the lack of DA transporter^4^. The COMT gene contains a functional polymorphism (Val158Met; rs4680). Because the Met alleles leads to a fourfold decrease in enzyme activity at body temperature, in the Val homozygotes individuals show the greater COMT activity and the lower dopamine level than the Met carriers^35^. There was two splice isoforms of DRD2: the short (D2S) isoform locates presynaptically and the long (D2L) isoform distributes postsynaptically^36^. SNP rs1076560 (G > T) within the DRD2 gene at intron 6 was associated with decreased expression of D2S relative to D2L^37^. In contrast to the association between GG genotype and more expression of D2S^38^, T-allele carriers are related to more expression of D2L^39^. Because the D2S isoform inhibits dopamine release^40^, subjects with DRD2 GG genotype predict lower DA signaling than T-allele carriers. Thus, subjects with Val/Val and GG genotypes may exhibit the lowest DA signaling and subjects with Met and TT genotypes may have the highest DA signaling.

The DMN consists of the posterior cingulate cortex, the lateral parietal cortex and the ventromedial prefrontal cortex, which plays an important role in human cognitive functions^41^,^42^,^43^. The structural and functional impairments of the DMN have been reported in several neuropsychiatric diseases, and these impairments have been associated with cognitive deficits^44^. In consistent with respective modulation effect of COMT or DRD2 on the MPFC connectivity^45^,^46^, we found a COMT×DRD2 interaction effect on intra-network connectivity in the left MPFC of the aDMN in healthy young subjects. The interaction was driven by different effects of DRD2 on connectivity between COMT genotypic subgroups. The DRD2 GG subgroup exhibited weaker intra-network connectivity than the GT subgroup in the COMT Val/Val carriers; however, this modulation is reversed in the COMT Met carriers. Our findings are also consistent with previously reported modulation effect of the dopamine-related genetic variations on DMN connectivity^8^,^47^. That is, Val/Val-TT or Met-GG carriers with optimal DA levels showed higher intra-DMN connectivity than Val/Val-GG with lower DA levels and Met-TT carriers with higher DA levels.

The SN mainly consists of the fronto-insular cortex (FIC) and the dACC^48^. The SN serves to identify salient stimuli around environment and to initiate control signals in time to coordinate functions of other cognitive-related networks^49^. In consistent with a previous imaging genetics study that reported a COMT×DRD2 interaction on the FCD of left dACC in healthy young adults^8^, we found an interaction effect on intra-network connectivity in the left dACC of the SN, which was driven by the lack of DRD2 modulation in the COMT Val/Val carriers and the strong modulation in the COMT Met carriers. As a critical component of the SN, the dACC was associated with decision-making and reward system^50^,^51^. This modulation effect of the DA-related genetic variations on the intra-SN connectivity may help for understanding the relationship between DA system and cognitive performance^52^,^53^.

The DAN including parts of the intraparietal cortex and superior frontal cortex, is involved in preparing and applying top-down selection for stimuli and responses^54^,^55^. Genetic variations in COMT and DRD2 have been associated with attention performance^56^,^57^ and attention bias^58^. In this study, we provided a possible pathway that DA-related genetic variation may affect human attention via modulating the intra-network connectivity of the DAN. Specifically, we found a COMT×DRD2 interaction on intra-network connectivity in the right dorsolateral frontal cortex of the rDAN, which was driven by the inversed modulation of DRD2 on connectivity in the COMT Val/Val and Met carriers.

The functional networks of the human brain have been divided into two functional systems: the “processing system” includes the visual, sensorimotor, and DMN; and the “control system” consists of the fronto-parietal, attention, and SN^34^. By investigating the interaction effects of COMT and DRD2, a previous study has revealed a functional system-dependent modulation of the DA signaling on FCD in healthy young subjects. Brain regions of the SN of the “control system” exhibit the highest FCD in DRD2 GG in the COMT Val/Val carriers and the lowest FCD in GG subgroup in the COMT Met carriers; however, brain regions of the visual network and DMN of the “processing system” showed an inverse modulation: the lowest FCD in DRD2 GG in the COMT Val/Val carriers and the highest FCD in GG in the COMT Met carriers^8^. In the present study, the modulation patterns on the SN and DMN connectivity are consistent with the hypothesis of functional system-dependent modulation. Because the DAN is thought to be a typical functional network of the “control system”^34^, the completely different modulation effects of dopamine-related genetic variations on intra-network connectivity in the DAN and SN of the “control system” may support a network-dependent modulation of the DA signaling on functionality of the brain. In contrast to significant differences on RSN connectivity, we did not found any significant differences in the cognitive, emotional, or personality scores among different genotypic groups. These findings may indicate that imaging measures are more sensitive than behavioral measures in detecting potential genetic effects in healthy young subjects. The lack of significant differences in behavioral performance across genotypic groups may also exclude the possibility that our imaging genetics findings are resulted from behavioral differences across genotypes.

Several limitations should be noted when one interprets our results. Although separating the subjects into different groups before applying GICA can remain the maximal differences across groups, this approach may bring mismatched components for different groups, which will create difficulty for intergroup comparisons. To avoid the need for matching components between groups, we performed GICA on combined data from both subject groups and then reconstructed subject-specific maps and time courses for group comparisons. However, the resulting ICA components tend to be similar among subjects, reducing the statistical power of the following ANCOVA. Another limitation of this study is the use of a relatively higher voxel-level threshold (*p* < 0.05) in the cluster-level correction for multiple comparisons. Thus, our results should be treated as preliminary and need to be further validated.

In conclusion, with a relatively large sample of healthy young adults and an ICA analysis, we explored the interaction effects of COMT and DRD2 on intra-network connectivity in resting-state networks. We found that a different modulation of DRD2 on intra-network connectivity in different COMT genotypic subgroups drove the interactions. Moreover, the effects DRD2 on connectivity in COMT subgroups were different across resting-state networks, suggesting that the nonlinear modulation of the dopamine-related genes on functionality of the brain is network-dependent.

## Additional Information

**How to cite this article**: Zhao, F. *et al*. Network-Dependent Modulation of COMT and DRD2 Polymorphisms in Healthy Young Adults. *Sci. Rep*. **5**, 17996; doi: 10.1038/srep17996 (2015).

## Supplementary Material

Supplementary Information

## Figures and Tables

**Table 1:** Demographic data of subjects in fMRI analysis (*n* = 250).

Genotypic groups	*n*	Age (years)	Years of education	Gender
COMT				
Val/Val	120	22.7 (2.4)	15.8 (2.0)	54:56
Met carriers	130	22.6 (2.4)	15.5 (2.1)	61:67
F (P)	250	0.11 (0.74)	0.14 (0.71)	0.49(0.49)
DRD2				
TT	39	22.8 (2.6)	15.7 (2.0)	15:13
GT	115	22.6 (2.3)	15.6 (2.2)	56:59
GG	96	22.7 (2.4)	15.8 (2.0)	44:51
F (P)	250	0.27 (0.76)	0.41 (0.66)	0.41(0.68)
COMT×DRD2				
Val/Val-TT	21	22.4 (2.4)	15.4 (1.9)	7:14
Val/Val-GT	56	22.3 (2.4)	15.7 (2.1)	28:28
Val/Val-GG	43	23.3 (2.3)	16.2 (2.0)	19:24
Met carriers-TT	18	23.4 (3.0)	16.1 (2.1)	8:9
Met carriers-GT	59	22.8 (2.3)	15.4 (2.3)	28:31
Met carriers-GG	53	22.1 (2.4)	15.4 (1.9)	25:27
F (P)	250	3.93 (0.02)	1.63 (0.20)	0.39 (0.68)

**Table 2:** Brain areas showed significant COMT×DRD2 interaction effects on brain networks.

Brain regions	RSN	Cluster size (voxels)	Peak intensity	MNI Coordinates (*x*, *y*, *z*)
Left medial prefrontal cortex	aDMN	75	6.5649	−15, 45, 33
Left dorsal anterior cingulated cortex	SN	93	7.3553	−3, 12, 33
Right dorsolateral frontal cortex	rDAN	60	10.9014	33, −15, 63

**Table 3:** Intergroup differences in intra-network connectivity in the MPFC of the DMN.

P values	Val/Val-GT	Val/Val-TT	Met carriers-GG	Met carriers-GT	Met carriers-TT
Val/Val-GG	0.013*	0.066	0.007*	0.494	0.693
Val/Val-GT		0.952	0.788	0.001**	0.145
Val/Val-TT			0.795	0.014*	0.238
Met carriers-GG				<0.001**	0.102
Met carriers-GT					0.357

*p < 0.05, uncorrected for multiple comparisons; **p < 0.05/15 = 0.0033, Bonferroni corrected for multiple comparisons.

**Table 4:** Intergroup differences in intra-network connectivity in the DLFC of the rDAN.

P values	Val/Val-GT	Val/Val-TT	Met carriers-GG	Met carriers-GT	Met carriers-TT
Val/Val-GG	<0.001**	0.175	0.097	0.242	0.724
Val/Val-GT		0.151	0.043*	<0.001**	0.002**
Val/Val-TT			0.936	0.020*	0.152
Met carriers-GG				0.0025**	0.107
Met carriers-GT					0.614

*p < 0.05, uncorrected for multiple comparisons; **p < 0.05/15 = 0.0033, Bonferroni corrected for multiple comparisons.

**Table 5:** Intergroup differences in intra-network connectivity in the dACC of the SN.

P values	Val/Val-GT	Val/Val-TT	Met carriers-GG	Met carriers-GT	Met carriers-TT
Val/Val-GG	0.499	0.092	0.014*	0.297	0.0266*
Val/Val-GT		0.224	0.053	0.699	0.005*
Val/Val-TT			0.817	0.346	0.001**
Met carriers-GG				0.114	<0.001**
Met carriers-GT					0.002**

*p < 0.05, uncorrected for multiple comparisons; **p < 0.05/15 = 0.0033, Bonferroni corrected for multiple comparisons.
