# Supplementary material for: Network-Dependent Modulation of COMT and DRD2 Polymorphisms in Healthy Young Adults
Source: Sci Rep. 2015 Dec 8;5:17996. doi: 10.1038/srep17996 (PMC4672286; doi:10.1038/srep17996)
Supplement: Supplementary Information [file srep17996-s1.doc]

**Supplementary Information**

To manuscript titled:

**“Network-Dependent Modulation of COMT and DRD2 Polymorphisms in Healthy Young Adults”**

By authors: Fangshi Zhao, Xuejun Zhang, Wen Qin, Feng Liu, Qiuhui Wang, Qiang Xu, Junping Wang, Chunshui Yu

In the supplementary material, we present the main effects of COMT and DRD2.

There were significant main effects of COMT and DRD2 on intra-network connectivity. We found a main effect of COMT on the intra-network connectivity in the right dorsolateral frontal cortex (*x* = 51, *y* = -15, *z* = 48) of the rDAN (*p* < 0.05, Alphasim correction). *Post-hoc* analysis showed that Met carriers exhibited significantly decreased intra-network connectivity than Val homozygotes (*p < 0.001*) (Figure S1). We also a main effect of COMT on the intra-network connectivity in the right dorsolateral prefrontal cortex (*x* = 27, *y* = 12, *z* = 42) of the rFPN (*p* < 0.05, Alphasim correction). *Post-hoc* analysis also showed significantly decreased intra-network connectivity in Met carriers than in Val homozygotes (*p < 0.001*) (Figure S2). There was a DRD2 main effect on the intra-network connectivity in the right dorsolateral prefrontal cortex (*x* = 18, *y* = 27, *z* = 45) of the rFPN (*p* < 0.05, Alphasim correction). *Post-hoc* analysis revealed a significant allele-dependent effect (TT > GT> GG) on the intra-network connectivity (TT > GT: *p = 0.518;* TT > GG: *p < 0.001;* GT> GG: *p < 0.001*) (Figure S3).


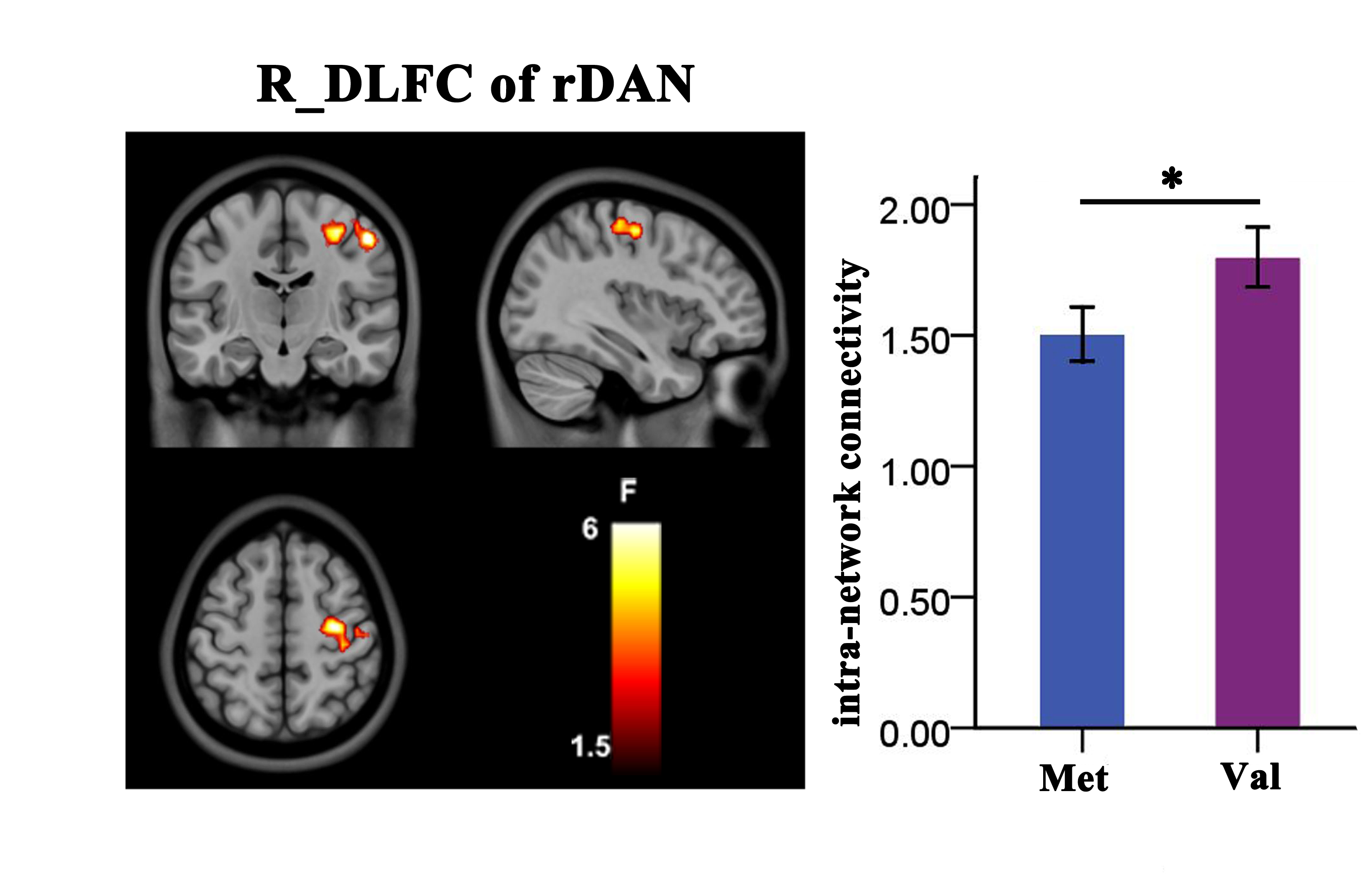


**Figure S1.** The main effect of COMT on the intra-network connectivity of the right dorsal attention network (rDAN). R, right. DLFC, dorsal lateral frontal cortex. * represents *p* < 0.05.


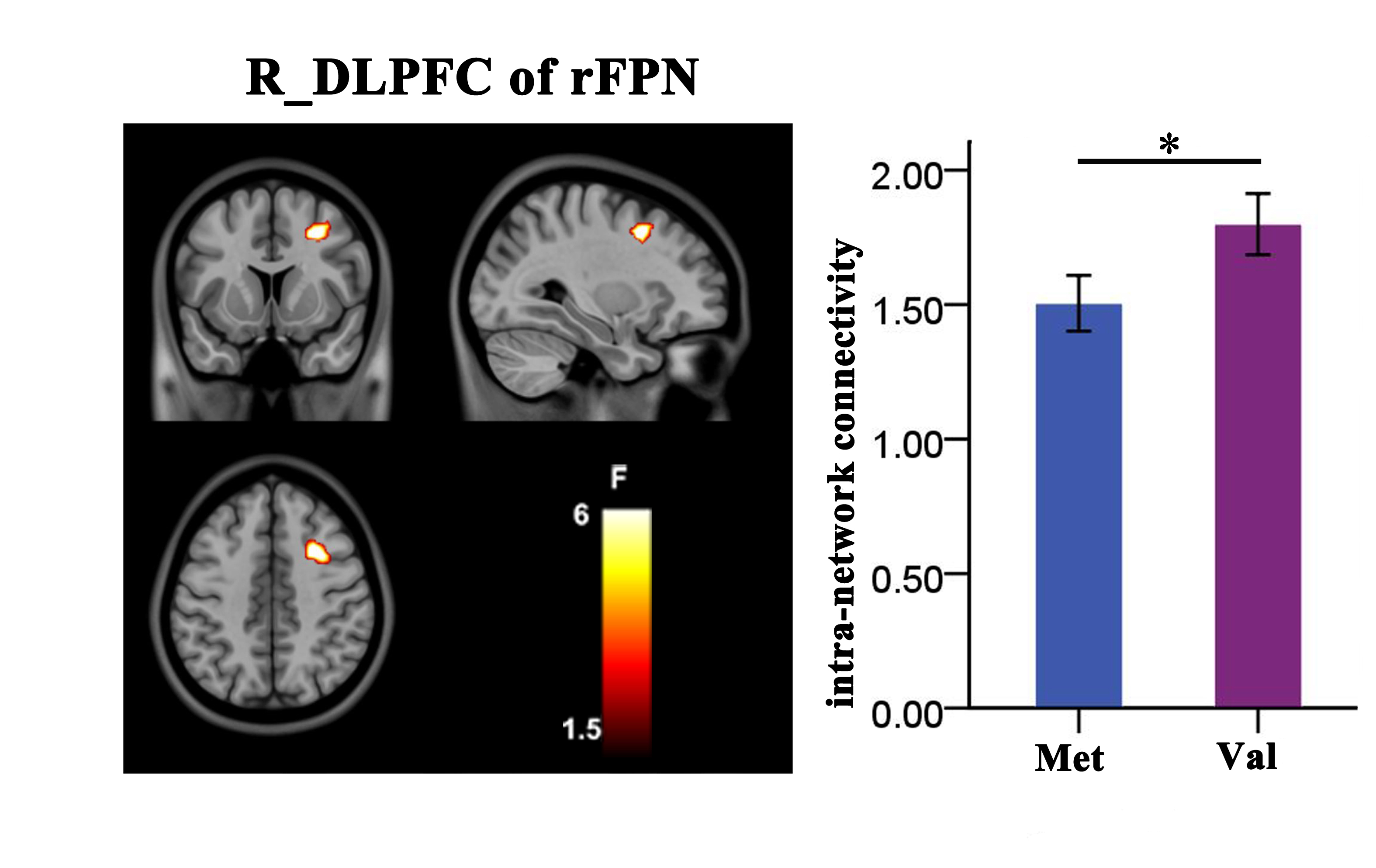


**Figure S2.** The main effect of COMT on the intra-network connectivity of the right fronto-parietal network (rFPN). R, right. DLPFC, dorsal lateral prefrontal cortex. * represents *p* < 0.05.


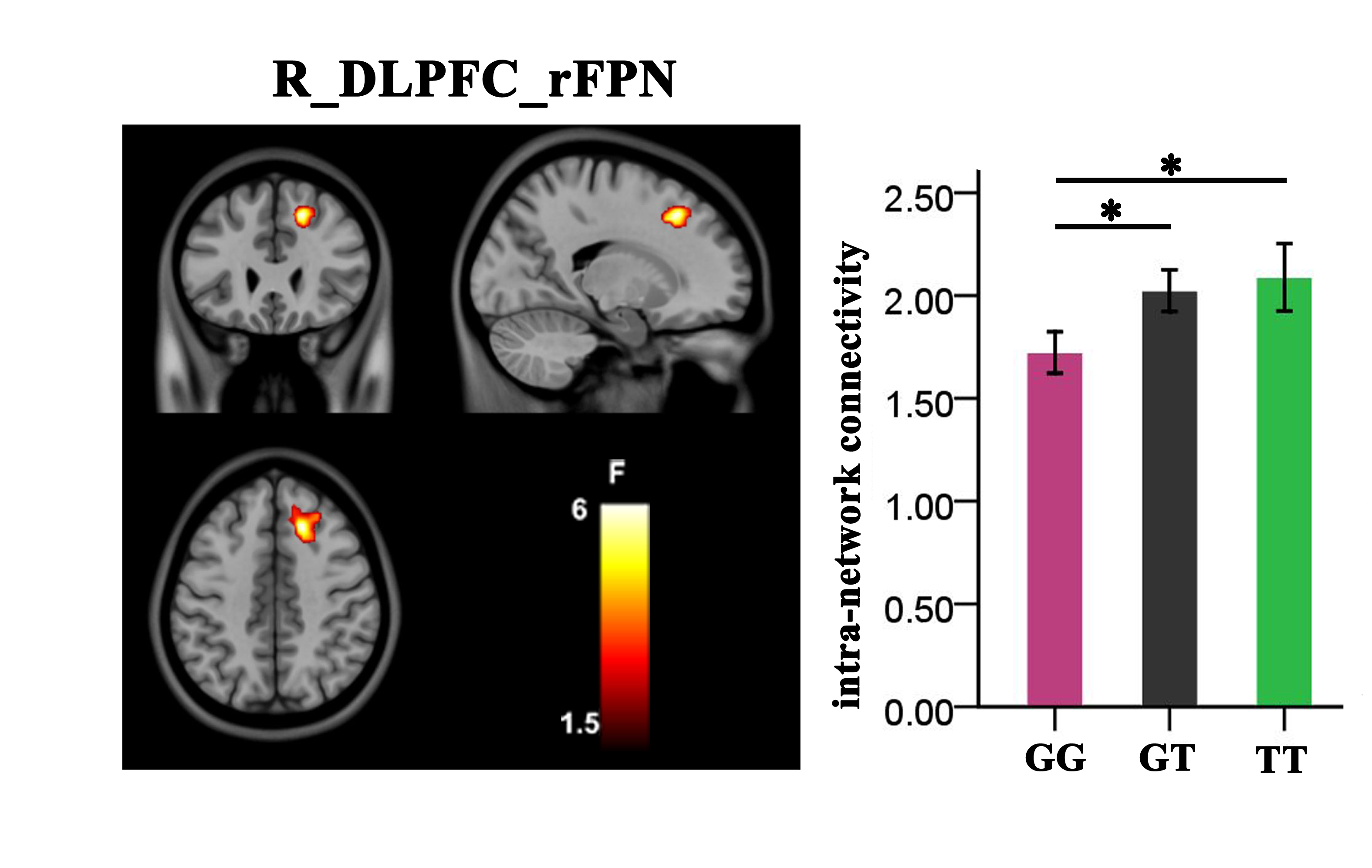


**Figure S3.** The main effect of DRD2 on the intra-network connectivity of the right fronto-parietal network (rFPN). R, right. DLPFC, dorsal lateral prefrontal cortex. * represents *p* < 0.05.
